# Mouse Models for Unravelling Immunology of Blood Stage Malaria

**DOI:** 10.3390/vaccines10091525

**Published:** 2022-09-14

**Authors:** Adesola C. Olatunde, Douglas H. Cornwall, Marshall Roedel, Tracey J. Lamb

**Affiliations:** Department of Pathology, University of Utah, Emma Eccles Jones Medical Research Building, 15 N Medical Drive E, Room 1420A, Salt Lake City, UT 84112, USA

**Keywords:** mouse models, *Plasmodium*, adaptive immunity, innate immunity, T cells, B cells, macrophages, neutrophils, antibodies, cytokines

## Abstract

Malaria comprises a spectrum of disease syndromes and the immune system is a major participant in malarial disease. This is particularly true in relation to the immune responses elicited against blood stages of *Plasmodium*-parasites that are responsible for the pathogenesis of infection. Mouse models of malaria are commonly used to dissect the immune mechanisms underlying disease. While no single mouse model of *Plasmodium* infection completely recapitulates all the features of malaria in humans, collectively the existing models are invaluable for defining the events that lead to the immunopathogenesis of malaria. Here we review the different mouse models of *Plasmodium* infection that are available, and highlight some of the main contributions these models have made with regards to identifying immune mechanisms of parasite control and the immunopathogenesis of malaria.

## 1. The Immune Response to *Plasmodium* Asexual Blood Stages Dictates Malarial Disease

Malaria is still a significant problem in the world with over 600,000 deaths resulting from 241 million cases in 2021, 95% of them concentrated in the African subcontinent [1]. The RTS, S Mosquirix™ vaccine in children has limited efficacy [2,3] but current efforts in improving this vaccine appear to be moving towards vaccine-mediated protection that is more durable [4]. Nonetheless, any further improvements in the development of efficacious therapeutics and vaccines require a better understanding of what constitutes an effective anti-malarial immune response.

Malaria is caused by infection with parasites of the species *Plasmodium*, deposited into the dermis of the skin by female *Anopheles* mosquitos while probing for a blood meal. The sporozoites travel through the blood circulation, invading the liver where they undergo several rounds of asexual division in hepatocytes before being released as merozoites into the blood stream. Whilst hepatocytes infected with *P. falciparum* always transition to liver schizogony, some species such as *P. vivax* and *P. ovale* can also differentiate to become a dormant stage known as a hypnozoite that can reactivate to cause malaria relapses [5]. Upon release into the blood stream parasites infect red blood cells (iRBCs) and replicate every 24–72 h depending on species. Clinical symptoms of malaria are exclusively caused by the erythrocytic lifecycle of *Plasmodium*.

The clinical manifestations of malaria are wide-ranging and include symptoms such as hypoglycemia, acidosis and anemia. Accumulation and sequestration of iRBCs on vascular endothelial cells is associated with vascular activation which is known to underlie organ-specific pathologies such as cerebral malaria, acute lung injury, hepatomegaly and liver fibrosis [6] (Table 1). While sterile immunity to malaria generally does not occur, years of repeated exposure to the parasite in endemic regions facilitates the development of clinical immunity that can be characterized by reduced parasite load (anti-parasite immunity) and controlled inflammatory responses to iRBCs (clinical immunity). Malarial disease encompasses a spectrum of virulence which is influenced by a number of factors including genetic variation of both the host and parasite [7,8,9], the make-up of the intestinal microbiome of the host [10] and environmental influences such transmission intensity or the presence of co-infections [11,12,13].

**Table 1:** The main clinical manifestations of *Plasmodium* infection.

Disease Manifestation	Species of *Plasmodium*	Probable Mechanism	Severity	References
Fever	All species	Schizogony-induced inflammation from 24–72 h depending on parasite species. Mediated by endogenous pyrogens induced during schizogeny such as TNF-α, IL-6, IL-1β and prostaglandin E_2_).	Not generally lethal	[14]
Anemia	All species	Erythrophagocytosis. Dyserythropoiesis. RBC destruction by parasite replication.	Can be lethal	[15]
Cerebral malaria	*P. falciparum* (children) *P. falciparum* and *P. vivax* (Adults)	Vascular activation via parasite sequestration on the brain endothelium, followed by breakdown of the blood brain barrier, vasogenic odema and herniation.	20% mortality	[16,17,18,19]
Malaria-associated acute respiratory distress and Malaria-associated Acute Lung Injury	*P. falciparum*, *P. vivax* *P. knowlsei* *P. ovale*	Vascular activation via parasite sequestration on the pulmonary endothelial followed by pulmonary leak.	40% mortality	[20,21,22]
Hepatomegaly/Liver fibrosis	*P. falciparum* *P. vivax* *P. ovale*	Jaundice and hepatic dysfunction due to infiltration of iRBCs and sequestration of iRBCs in the liver. Results in activation of hepatic stellate cells to become myofibroblasts.	Normally an indicator of severe malaria	[23,24]
Acute Kidney Injury (AKI)	*P. falciparum* *P. vivax* *P. malariae*	Glomerulonephritis, acute tubular necrosis and acute interstitial nephritis due to hemodynamic dysfunction and inflammation. Results in proteinuria, microalbuminuria and urinary casts along with hemolytic-uremic syndrome. Contributes to metabolic acidosis and can be exacerbated by liver damage.	Normally an indicator of severe malaria and found in around 40% of those with severe disease	[25]
Lactic acidosis	*P. falciparum*	Tissue hypoperfusion and hypoxia resulting from capillary obstruction with sequestered iRBCs and anemia Production of lactate by iRBCs. Impaired lactate clearance by the liver and kidney.	Normally an indicator of severe malaria	[26]
Hypoglycemia	*P. falciparum* *P. vivax*	Illness-induced fasting and inhibition of gluconeogenesis.	An indicator of severe malaria and more common in children than adults. Predicts mortality in malaria	[27]

Mouse models are commonly used to study the immunology of erythrocytic malaria. Given the well-characterized range of different rodent *Plasmodium* species and strains, as well as the plethora of mouse lines currently available to investigators, this article will outline some of the parasite-mouse combinations that are commonly used to study the different facets of blood stage malaria immunology. In addition, we will discuss novel models of rodent malaria that have not yet been fully harnessed to determine the environmental and genetic contributions to generating immune responses to *Plasmodium* iRBCs.

## 2. Utility of Rodent *Plasmodium* Species in the Investigation of Blood Stage Immunology

Human parasites cannot infect mice unless the mice are genetically humanized [28,29]. Whilst humanized mouse models have some utility in the investigation of immune responses to *P. falciparum* in a controlled environment, several species of *Plasmodium* exist that naturally infect rodents (Table 2). Isolated and cloned from *Thamnomys* thicket rats in the Central African Region in the 1960s [30], they have been instrumental in the study of the immunobiology of the erythrocytic stages of *Plasmodium* infection [31]. Although apparently asymptomatic in their original hosts, infection of mice gives rise to a number of different phenotypes of infection, many of which mimic various states of disease found in human *Plasmodium* infection. Rodent *Plasmodium* parasites cannot infect humans making them tractable and non-hazardous models of malaria. However rodent *Plasmodium* parasites have some differences to human *Plasmodium* parasites such as the variant antigen gene families expressed in the blood stage. Rodent *Plasmodium* parasites do not have *var* genes encoding *P. falciparum* erythrocyte membrane protein-1 (*Pf*EMP1) [32,33], nor genes encoding the subtelomeric variant open reading frame (STEVOR) [34] or *P. falciparum*-encoded repetitive interspersed families of polypeptides (RIFINS) [35]. Instead, rodent *Plasmodium* parasites rely on genes encoded by *Plasmodium* interspersed repeat (*pir*) genes [36,37,38,39] which are the largest multigene family in many *Plasmodium* species [39]. It is important to note that no single rodent *Plasmodium* species replicates all features of human *Plasmodium* infection. Therefore, specificity of the focus of a particular study in combination with the correct choice of model is a key aspect of research into blood stage malaria immunology using rodent models of malaria.

**Table 2:** Disease phenotype and pathophysiology of the main rodent *Plasmodium* species used in biomedical research.

Species	Clone	RBC Preference	Phenotypes	References
*P. berghei*	ANKA	Reticulocyte preference but will invade normocytes	Asynchronous life cycle, sequesters in the liver, lung and brain. Evidence of weight loss and anemia normally present at the time of death. C57BL/6J: lethal infection with breakdown of the BBB and death between day 7–10 p.i. Pronounced pulmonary pathology. BALB/c: Death from hyperparasitemia. No discernible cerebral complications. Less extensive lung pathology. DBA/2J: No discernible cerebral complications. some pulmonary pathology but less pronounced than BALB/c mice. Death ~day 20 p.i from hyperparasitemia and anemia. Pet shop mice: resistant to death by cerebral malaria. Death ~day 20 p.i. from hyperparasitemia.	[40,41,42,43]
	NK65 New York (NY)	Reticulocyte preference	Accumulates mostly in the lung, with very little accumulation in the brain. Causes anemia over the course of infection. C57BL/6: death in ~20 days from respiratory distress. BALB/c: no development of MA-ARDS.	[44,45]
	NK65 Edinburgh (E)	Normocytes and Reticulocytes	Accumulates in the lung but not the brain with some evidence of anemia. C57BL/6: death in 7–10 days from respiratory distress. Early increase in peripheral parasitemia. BALB/c: Resistant to respiratory distress upon infection.	[44,45]
	K173	Reticulocyte preference	Very little parasite accumulation/sequestration in the brain. Does not produce gametocytes due to laboratory adaptation from passaging. C57BL/6: Used as model for cerebral malaria. Early death after infection due to cerebral pathology accompanied with very high parasitemia. Causes lung pathology with increased pulmonary oedema.	[46,47]
*P. yoelii*	XL (also known as 17XL)	Normocytes and reticulocytes	C57BL/6: Lethal within ~10 days p.i. due to hyperparasitemia and severe anemia. BALB/c: Lethal within ~10 days p.i. DBA/2: Non-lethal infection.	[48]
	XNL (also known as 17XNL)	Strong reticulocyte preference	C57BL/6: Resolving non-lethal infection accompanied by anemia. BALB/c: Resolving non-lethal infection.	[48,49]
	YM	Normocytes and reticulocytes	C57BL/6: Derivative of the XL line. Lethal within ~10 days p.i. due to hyperparasitemia and severe anemia. DBA/2: Lethal infection in ~10 days p.i. B10: Non-lethal infection.	[50,51,52,53]
	*nigeriensis* N67	Normocytes and reticulocytes	C57BL/6: Lethal at ~15–20 days p.i. due to hyperparasitemia.	[50,52,54]
	*nigeriensis* N67C	Normocytes and reticulocytes	C57BL/6: Lethal within 7 days p.i.	[50,54,55]
*P. chabaudi*	*chabaudi* AS	Normocytes and reticulocytes	Synchronous life cycle. Sequesters predominantly in the lung and liver. C57BL/6: Resolving non-lethal infection accompanied by anemia, thrombocytopenia, hypoglycemia, weight loss and hypothermia. Recrudescent infections and sub-patent for up to 3 months. BALB/c: More severe infection than in C57BL/6J mice but generally non-lethal in most BALB/c lines. A/J mice: Lethal anemia due to poor control of iRBCs and hyperparasitemia.	[31,56]
	*chabaudi* BC	Normocytes and reticulocytes	C57BL/6: similar symptoms to *P. chabaudi* AS but more severe.	[57]
	*chabaudi* CB	Normocytes and reticulocytes	C57BL/6: similar symptoms to *P. chabaudi* AS but more severe.	[58]
	*chabaudi* ER	Normocytes and reticulocytes	C57BL/6: Similar symptoms to those of *P. chabaudi* AS. Recrudescent infections occurs at 20 to 25 days p.i.	[31,57,59]
	*chabaudi adami*	Preference for younger normocytes over reticulocytes	BALB/c mice: Non-lethal infection with single peak of infection around 10 days p.i. A/J mice: Non-lethal resolving infection. C57BL//6 mice: Non-lethal resolving infection.	[60,61,62,63,64]
*P. vinckei*	*vinckei* CY	Normocytes Not thought to invade reticulocytes	CBA: Lethal infection by 6 days p.i. with hyperparasitemia.	[65]
	*vinckei* ATCC 30091	Normocytes Not thought to invade reticulocytes	ICR outbred mice: Lethal infection within 8–10 p.i.	[66]
	petteri AS	Normocytes Not thought to invade reticulocytes	AKR mice: Lethal in 5 days p.i. due to fast growing parasites.	[67,68]
	petteri BS	Normocytes Not thought to invade reticulocytes	ICR outbred mice: Non-lethal infection with a peak of parasitemia at 9 days p.i. CBA: Non-lethal infection with a peak of parasitemia at 9 days p.i.	[65,66]
	*petteri* AR	Normocytes Not thought to invade reticulocytes	AKR mice: Non-lethal with patent parasitemia not detectable by 22 days p.i.	[67,68]
	*petteri* CR	Normocytes Not thought to invade reticulocytes	BALB/c: single peak of non-lethal infection. CBA: single peak at 6 days p.i.; non-lethal infection.	[65,69]
	*petteri* HW	Normocytes Not thought to invade reticulocytes	C57BL/6: Lethal infection at 8–10 days p.i. from hyperparasitemia.	[70]

Abbreviations: BBB: blood-brain barrier; MA-ARDS: malaria-associated acute respiratory distress syndrome; p.i.: post-infection.

Mice have been instrumental in elucidating the workings of the human immune system [71]. Nonetheless, there are fundamental differences between the physiology of mice and humans that should be noted such as a different balance of leukocyte subsets [72] between both species as well as in splenic architecture where human (sinusoidal) and mouse (non-sinusoidal) [73] differences would alter the direction of blood flow and possibly the timing or mechanisms by which iRBCs induce splenic immune responses [73,74]. Splenic sinusoids are blood vessels in the red pulp that drain into the white pulp of the spleen where most splenic immune cells reside. In humans the endothelial cells lining these sinuses form a barrier that RBCs have to squeeze through [74,75], a challenge for *Plasmodium*-iRBCs which are more rigid than uninfected RBCs. On the other hand, the mouse spleen endothelial cells have bigger gaps making it more likely for iRBCs to end up in the white pulp with a potentially expedited adaptive response that may be based on a higher antigenic load compared to humans with *Plasmodium* infection. Human spleens also lack a substantial marginal zone [73], a B-cell enriched layer that also contains unique macrophage populations that surrounds the follicles and periarteriolar sheath separating the white and red pulp.

Circulating *Plasmodium* parasites have been found to alter expression of variant antigens expressed on the RBC surface in both splenectomized squirrel monkeys infected with *P. falciparum* [76] and splenectomized mice infected with *P. chabaudi* [77] suggesting a splenic response occurs in primates and rodents infected with *Plasmodium.* Disorganization of the white pulp in both human autopsy samples of malaria fatalities [78] and in mice infected with *P. chabaudi* [79,80] also suggests involvement of the spleen in both species. However, the structural differences that exist may impact the timing of immunological events and possibly the importance of different cell types in orchestrating the immune response. As such, some caution should be exercised when extrapolating findings from rodent *Plasmodium* blood stage models with respect to the main players involved.

**Figure 1:**
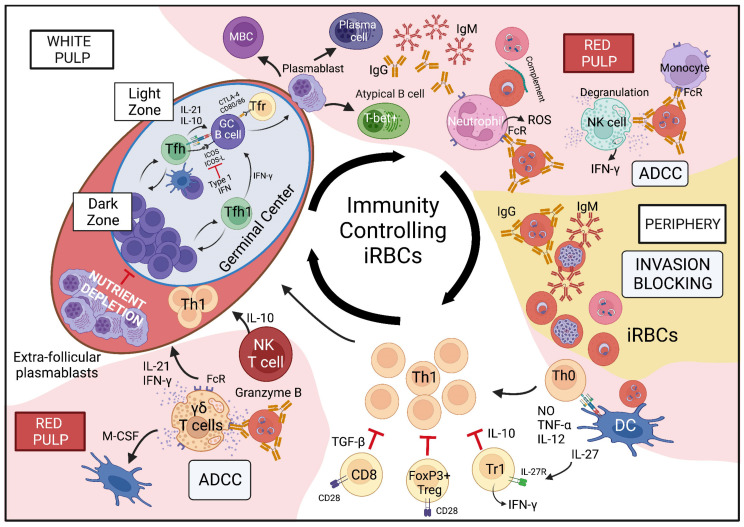
Contribution of mechanisms influencing immune control to *Plasmodium*-infected red blood cells. Mouse models of malaria have been used to demonstrate how innate and adaptive immune mechanisms synergize to effectuate control of iRBCs. In the white pulp of the spleen activation of T helper cells including Th1 cells, Tfh cells and regulatory cells (both Treg cells and Tr1 cells) are activated by antigen presenting cells which have phagocytosed iRBCs by-products of parasite development that are released during schizogony. Tfh cells are critical in providing help to B cells to produce anti-parasite antibodies which play important roles via interaction with innate cells antibody-dependent cellular cytotoxicity and in invasion blocking upon schizogony. The generation of anti-parasite antibodies both within and out-with germinal centers has been interrogated in mouse models of malaria gleaning important contributions towards what constitutes an efficacious cellular response generating efficacious antibodies reactive against iRBCs. Figure created with Biorender. Abbreviations: ICOS: inducible T cell costimulatory; IFN: interferon; Ig: immunoglobulin; IL: interleukin; iRBC: infected red blood cell; LLPC: long-lived plasma cell; M-CSF: macrophage colony-stimulating factor; NO: nitric oxide; ROS: reactive oxygen species; TGF: transforming growth factor; TNF: tumor necrosis factor.

Despite these differences, the main features of the immune response to *Plasmodium* iRBCs (Figure 1, Table 3) are largely replicated with a strong inflammatory response characterized by interferon-γ (IFN-γ) producing CD4 T cells and the production of anti-parasite antibodies. Mouse models of *Plasmodium* infection provide a tractable and highly informative model to define how the immune system operates in human *Plasmodium* infection, in turn providing critical evidence of immune mechanisms in malaria that simply cannot be obtained in humans. Advancements in both rodent genetic engineering technology [81,82] and the ability to create transgenic rodent *Plasmodium* parasites [83,84] (Table 4) has facilitated dissection of immune responses to *Plasmodium* infection with unprecedented precision. As such, mouse models of blood stage *Plasmodium* infection are a key tool in understanding the immune responses driving *Plasmodium* parasite control and pathogenesis of malaria.

Selection of a mouse host and parasite species to study immune responses to blood stage *Plasmodium* infection is dependent on the question being asked. Some mouse-parasite combinations are lethal from around 7 days post-infection whereas others resolve to become a sub-patent infection that can only be detected by molecular methods and, in some cases, can be completely cleared. When selecting which combination to use it is important to determine whether the major goal of any study is to decipher anti-parasite immune responses, clinical immunity or a combination of both. Other considerations may involve the existence of comparative literature, the availability of transgenic tools (Table 4) or the existence of databases from “big data” sets available online (Table 5) that can be mined *a priori* to identify candidate molecules of importance.

**Table 3:** The main immunological features of the three most widely used *Plasmodium* species in the study of the immune response elicited by *Plasmodium*-infected RBCs.

Rodent Species		Model Uses	Main Features	References
*Plasmodium chabaudi*		Innate immune responses	Control of iRBCs by monocytes and γδ T cells, but not neutrophils or NK cells. Direct activation of DC for activation of T cell responses.	[85,86,87,88,89,90,91,92]
		T cell responses	Requires T cells for control of iRBCs.	[59]
		Immune regulation	IL-10 is required for clinical immunity. TGF-β provides some protection against pathogenesis.	[93,94]
		Generation of humoral immunity	Participation of both IgG and IgM in control of iRBCs. Does not require antibodies for control of acute infection with iRBCs. Antibodies contribute to control of iRBCs during chronic infection.	[11,95,96,97]
		T and B cell memory responses	Generates memory T and B cell responses that expand upon secondary challenge infection.	[98,99,100]
		Immune basis of clonal virulence	Clonal virulence is associated with differences in the immune response induction.	[101,102]
		Host genetic basis of immune resistance to infection by iRBCs	Genetic control of host immune responses mediates immunological control of iRBCs and level of clinical immunity.	[103,104]
*Plasmodium yoelii*		Innate immune responses	Macrophages are protective against iRBCs. Minimal contribution of neutrophils,γδ-T cells or NK cells to control of iRBCs.	[105,106,107]
		T cell responses	Requires T cell for control of iRBCs.	[108]
		Immune regulation	IL-10 and TGF-β are required for clinical immunity.	[93,109]
		Generation of humoral immunity	Requires antibodies for control of iRBCs during acute infection.	[110]
		T and B cell memory responses	Generates memory T and B cell responses that expand upon secondary challenge infection.	[111,112]
		Immune correlates of lethal vs. non-lethal infection	Lethality is correlated with faster parasite growth and an early burst of TGF-β.	[113]
*Plasmodium berghei*	ANKA	CD8 T cell induced vascular leak		[114,115]
		Pulmonary vascular leakage and leukocyte infiltration		[116]
		Clonal differences in the induction of experimental cerebral malaria		[117]
	NK65 Edinburgh	Pulmonary vascular leakage and leukocyte infiltration		[45,118]
	NK65 New York	Pulmonary vascular leakage and leukocyte infiltration		[45]

**Table 4:** Functionality of selected transgenic rodent *Plasmodium* parasites and mouse lines available to study of the immune response to *Plasmodium*-iRBCs. Repositories of rodent malaria strains available for use include the Rodent Malaria genetically modified Data Base (RMgmDB) (https://www.pberghei.eu/index.php), the European Malaria Reagent Repository (bank: http://www.malariaresearch.eu/) and the Malaria Research and Reference Reagent Resource Center (MR4) (https://www.beiresources.org/ProgramInformation.aspx). All links last accessed on 8 September 2022.

Transgene	Functionality	Transgenic Parasites	Properties of the Transgenic Parasite	References
Luciferase	In vivo and ex vivo visualization of organ-specific parasite sequestration. Can be imaged after injection of D-luciferin using an IVIS imager	*P. berghei* ANKA	Expression of luciferase under the eEF1α promoter (constitutive) or the AMA-1 promoter (schizont-specific).	[119]
		*P. berghei* NK65 Edinburgh	Expression of luciferase under the AMA-1 promoter (schizont-specific).	[118]
		*P. chabaudi* AS	Expression of luciferase under the eEF1α promoter (constitutive).	[56]
Allelic replacement of rodent *Plasmodium* proteins with human *Plasmodium* proteins	Study of immune responses to human *Plasmodium* parasite vaccine targets	*P. berghei* ANKA	Express *P. falciparum* merozoite protein-1_19_ (MSP-1_19_).	[120]
		*P. berghei* ANKA	Express *P. falciparum* apical membrane antigen-1 (AMA-1).	[121]
		*P. berghei* ANKA *P. berghei* NK65	Express *P. vivax* merozoite protein-1_19_ (MSP-1_19_).	[122]
Fluorescent proteins	Imaging of parasites ex-vivo by microscopy. Assessment of phagocytosis by flow cytometry or Imagestream.	*P. chabaudi AS*	Expression of GFP under the eEF1α promoter.	[85]
		*P. chabaudi* AJ	Expression of RFP under the eEF1α promoter.	[83]
		*P. yoelii* XNL	Expression of GFP under the eEF1α promoter.	[123]
		*P. berghei* ANKA	Expression of GFP under the eEF1α promoter.	[124]
		*P. berghei* NK65E	Expression of GFP under the AMA-1 promoter (schizont-specific).	[118]
		*P. berghei* NK65NY	Expression of GFP under the AMA-1 promoter (schizont-specific).	[45]
Insertion of peptide epitopes recognized by available TCR transgenic mice	Quantification of antigen-specific *Plasmodium*-induced T cells responses	*P. yoelii* XNL	Express a CD4 and CD8 immunodominant epitope from the glycoprotein LCMV. Can be tracked using SMARTA or P14 TCR transgenic mice.	[112]
		*P. yoelii* XNL	Express a CD4 and CD8 immunodominant epitope from the model antigen chicken ovalbumin. Can be tracked using OVA TCR transgenic mice (OT-I and OT-II).	[125]
		*P. berghei* ANKA	Express a CD4 and CD8 immunodominant epitope from the model antigen chicken ovalbumin. Can be tracked using OVA TCR transgenic mice (OT-I and OT-II).	[126]
**Endogenous parasite target epitope**	**Parasite species**	**Transgenic Mouse line**	**Properties of the transgenic mouse line**	**References**
RPL6_120–127_	*P. berghei* ANKA	Pb-I H2-K^b^ C57BL/6	Transgenic Pb-I CD8 T cells with Vα8.3/Vβ10 TCRs that recognize *Pb*RPL6_120–127_ in *P. berghei* ANKA liver stages but cross-react with blood stage antigens in *P. berghei* ANKA, *Pc*RPLL6_130–137_ (*P. chabaudi*) and *Py*RPL6_123–130_ (*P. yoelii*).	[127,128]
HSP90_484–496_	*P. berghei* ANKA	Pb-II I-A^b^ C57BL/6	Transgenic Pb-II CD4 T cells with Vα2/Vβ12 TCRs that react cross-react to *P. berghei* NK65, *P. chabaudi* AS and *P. yoelii* XNL.	[129,130]
37/39 kDa fragment of MSP-1_1157–1171_	*P. chabaudi* AS	B5 I-E^d^ BALB/c	Contains B5 MSP-1-reactive CD4 T cells with Vα2/Vβ8 TCRs reactive to MSP-1_1157–1171_.	[131]
21 kDa fragment of MSP-1	*P. chabaudi* AS	Igh^NIMP23/+^ C57BL/6	B cells express the NIMP23 Ig heavy chain and harbor B cells that react to the 21 kDa fragment of *P. chabaudi* AS MSP-1.	[132]

Abbreviations: AMA-1: apical membrane antigen 1; eEF1: eukaryotic elongation factor; GFP: green fluorescent protein; HSP: heat shock protein; LCMV: lymphocytic choriomeningitis virus; MSP-1: merozoite surface protein 1; RFP: red fluorescent protein; RPL6: putative ribosomal protein L6.

**Table 5:** Published and publicly available databases of sequencing data from rodent *Plasmodium* infections.

Cell Type	Mouse Strain	Parasite Strain	Sequencing Type	Database Access Code	References
Whole Blood and spleen	C57BL/6	*P. chabaudi chabaudi* AS and *P. chabaudi chabaudi* CB	Microarray	GSE93631	[133]
Whole Blood and spleen	C57BL6	*P. chabaudi chabaudi* AS	Microarray	GSE123391	[133]
				GSE145781	[134]
Bone marrow	C57BL/6J and C57BL/6J Elf4−/− mice	*P. yoelii* XNL	RNAseq	GSE121035	[135]
Monocyte derived dendritic cells	C57BL/6	*P. berghei* ANKA	RNAseq	GSE126381	[136]
Splenic Macrophages	C57BL/6	*P. berghei* ANKA	Microarray	GSE111593	[137]
Macrophages	BALB/c	*P. yoelii* XNL-Luc	RNAseq	GSE115906	[138]
Red pulp macrophages	C57BL/6	*P. chabaudi chabaudi* AS	Microarray	GSE23565	[139]
NK cells	C57BL/6	*P. chabaudi chabaudi* AS	Microarray	GSE12727	[140]
γδ T cells	C57BL/6J	*P. chabaudi chabaudi* AJ	Single cell TCR sequencing and RNAseq	GSE108478	[89]
CD4 T cells	C57BL/6	*P. berghei* ANKA	microarray	GSE24903	[141]
	C57BL//6J and Uba3^fl/fl^-Lck Cre+ (KO) Uba3^fl/fl^ and Uba3ΔT mice on a C57BL/6J background	*P. yoelii* XNL	RNAseq	GSE111066	[142]
	C57BL/6	*P. chabaudi chabaudi* AS	Microarray Single-cell RNAseq	GSE81196 GSE81197	[143]
	C57BL/6	*P. yoelii* XNL	Microarray	GSE85896	[144]
Regulatory T cells	BALB/c	*P. yoelii* XNL	Microarray	GSE34621	[49]
B cells	Tbx21fl/flCd23Cre and CD23Cre+ mice on a C57BL/6J background	*P. berghei* ANKA	RNAseq ATACseq	GSE120729 GSE120727	[145]
	IFN-γR1−/− and C57BL/6J	*P. chabaudi chabaudi* AS	RNAseq	GSE85205	[146]
	C57BL/6	*P. yoelii* XNL	RNAseq	GSE134548	[110]
	C57BL/6	*P. chabaudi chabaudi* AS	RNAseq	GSE115155	[132]
Microglia	C57BL/6 wildtype and IFNAR−/−	*P. berghei* ANKA	Microarray	GSE119650 GSE86082	[147]

Abbreviations: Cre: cre recombinase; Elf4: E74 Like ETS Transcription Factor 4: IFNAR: interferon-α receptor 1; IFN-γR1: interferon-γ receptor 1; Tbx21: T box transcription factor 21; Uba3: Ubiquitin-Activating Enzyme 3.

## 3. Genetic Control of the Immune Responses to *Plasmodium* Infection

Population-wide genetic diversity and its effect on *Plasmodium* infection is evident in human populations. This can be clearly seen with hemoglobinopathy gene polymorphisms such as sickle cell, thalassemia or glucose-6-phosphate dehydrogenase (G6PD) [148,149,150,151,152] which offer resistance to infection of RBCs by *Plasmodium* and reduced clinical severity when infection does occur. Associations have been found with MHC haplotype [153,154] with varying results [155], and polymorphisms in immune genes and the promoters controlling their expression have been associated with malarial disease severity. For example, allele variants [156] and promoter polymorphisms [157,158] controlling the expression of the inflammatory cytokine tumor necrosis factor-α (TNF-α) have been associated with the propensity to develop cerebral malaria [157,158] and anemia [157,159].

There is now an increasing number of publications using Genome-Wide Association Studies (GWAS) for malaria [160,161,162,163]. These studies have found associations of polymorphisms encoding an erythrocyte calcium pump (ATP2B4) and an endothelial junction protein (MARVELD3) with severe malaria [162], and linkages to genes on chromosome 6q21.3 and possibly 19p13.12 to the development of uncomplicated (mild) malaria [164]. Linkages to asymptomatic malaria have been found on chromosome 5q31 [164]. Collectively these data suggest that the development of immune responses during *Plasmodium* infection is, in part, genetically controlled. In support of this notion, the Fulani tribe of western Africa who are generally more resistant to the clinical effects of *Plasmodium* infection have allelic variants of FcγRIIα [165], interleukin (IL)-10 [166] and IL-4 [167] which are not present in the more susceptible sympatric Dogon tribe [168,169]. It is thought that these variants allow the Fulani to mount a robust and protective immune response to *Plasmodium* that is characterized by early production of pro-inflammatory cytokines like IFN-γ [170].

In mice *Plasmodium* infections are also genetically controlled [171,172,173,174]. Between-strain genetic diversity can explain the variation in disease severity in *Mus musculus* infected with any of the rodent *Plasmodium* lines. For example, it is widely accepted that C57BL/6 mice are susceptible to *P. berghei* ANKA (*Pb*A) infection succumbing to neurological manifestations of infection that resemble cerebral malaria, whereas BALB/c or DBA/2J are more resistant and survive significantly longer, dying much later from hyperparasitemia [173]. Linkage studies of *P. chabaudi*-AS-infected crossed lines of inbred, recombinant inbred and congenic inbred lines of mice has led to the identification of several gene regions termed Char (Chabaudi resistance) regions (reviewed in [103] and [104]) which include immune-associated genes such as tumor necrosis factor (TNF) and lymphotoxin-α (LT-α) amongst others demonstrating the importance of immunogenetics in the outcome of *P. chabaudi* infection in mice.

The majority of studies elucidating immune responses to the erythrocytic stage of *Plasmodium* infection are undertaken in genetically-uniform inbred mouse strains, many using mice with C57BL/6 and BALB/c backgrounds (Table 2). Whilst beneficial by virtue of uniform background genetics and MHC haplotype, such mice will not directly inform on the immunogenetic basis of disease severity. Although necessary with respect to studies incorporating genetically modified mouse lines, collectively such studies may bias our understanding of infection immunology due to their highly selected life histories in laboratory settings [175]. Many of these inbred strains have skewed immune responses, such as the Th1 skewed response in C57BL/6 mice or Th2-skewed response in BALB/c mice [176], which have been exploited to understand genetic influences on the differential immune responses mounted to *Plasmodium* parasites and the severity of infection [172]. As such, it is important to be cognizant of this limitation of mouse models when interpreting data that have been collected.

Several studies have endeavored to use outbred mouse lines, with Swiss Webster mice commonly used. Nonetheless, such mice have inbreeding coefficients of ~0.48 [177] which is rather removed from humans which are ~0.01–0.08 [178,179]. New endeavors to generate mouse resources that are more aligned with human diversity include wild-derived specific pathogen free (SPF) mice [180], or the Collaborative Cross (CC) mice [181,182,183,184] and Diversity Outbred (DO) mice [182,185,186]. These colonies allow assessments of the variation of immune responses to *Plasmodium* infections that may be more akin to humans. Given that all of these under-utilized mouse colonies are SPF, they have the advantage of assessing genetic diversity on anti-*Plasmodium* immune responses in the absence of contributing environmental factors.

## 4. Modelling the Influence of Environmental Factors on Immune Responses to *Plasmodium* Infection

Genetic diversity in immune responsiveness has been studied in wild-captured mice. However responses in such mice are confounded by environmental factors [187] which include differences in microbiota [188], a community of microorganisms including bacteria, fungi, viruses and protozoans. These microorganisms colonize a number of external facing environments of humans such as the respiratory tract, gastrointestinal tract and skin. Studies in humans and mice have found associations between gut microbiota and the susceptibility to *Plasmodium* infection [189,190]. Normally dominated by members of the *Firmicutes* and *Bacteroides* taxa, the diversity of the bacterial microbiota has been shown to increase upon *P. falciparum* infection [191,192]. Furthermore, upon infection with *Plasmodium* the level of malarial disease may be affected by the increased composition of bacterial microbiota. Specifically, it has been shown that severe malarial anemia in *P. falciparum*-infected children in Uganda is associated with a greater alpha diversity of bacteria in the gut [191].

Mouse models of malaria have been instrumental with respect to parsing out possible mechanisms underlying the effects of bacterial microbiota composition on immune responses to *Plasmodium* blood stages. In general, bacterial microbiota play a key role in glycan metabolism in the gut giving rise to monosaccharides that are fermented to short-chain fatty acids and used as an energy source for the host, but also have immunomodulatory functions. Differences in the metabolic capacity of the bacterial microbiome correlate with disease susceptibility to *P. yoelii* XNL infections [193]. Evidence that this may result from the immunomodulatory effects of the bacterial microbiome comes from studies showing that mice that are more resistant to *P. yoelii* XNL have microbiomes enriched for *Lactobacillus* and *Bifidobactierum* [10] and a better humoral response to *P. yoelii* XNL with a higher magnitude of anti-parasite antibodies produced. Germinal Center (GC) reactions to *Plasmodium* infection, as discussed below, are a key event resulting in an efficacious anti-*Plasmodium* humoral response. Highly dynamic in nature, splenic GC reactions have been shown to be continuously modulated by gut microbiota in *P. yoelii* XNL infections [191,194] demonstrating the utility of mouse models with regards to dissecting mechanisms by which microbiota may influence malarial disease in those infected with *Plasmodium*. The implications of this work for those undertaking research using mouse models of malaria to dissect the immunology of blood stage infections is the choice of mouse vendor; the severity of erythrocytic *Plasmodium* infection in genetically similar mice obtained from different vendors is significantly altered in response owing to the differences in gut microbiome [10].

Inbred mice that are removed from an SPF environment and have been exposed to natural environments (“re-wilded mice”) are found to have a different immune landscape modulated by the microbiota [188,195]. Recent work has studied the role of genetic diversity in *Plasmodium* immune responses in the context of environmental exposures through co-housing specific pathogen free (SPF) mice with mice obtained from pet shops that were not SPF (so-called “dirty mice”) [196]. Influencing the environmental exposure of mice in this way induced a less susceptible phenotype to *P. berghei* ANKA infections but did not alter immune responses sufficiently to fully protect all mice [196]. However, it should be noted that mice obtained from pet shops are highly inbred and do not recapitulate the genetic diversity conferred by wild-derived, CC or DO mice described above.

In addition to the microbiota, there are other environmental factors that collectively can influence the immune responses to *Plasmodium* iRBCs that are hard to consistently replicate in laboratory mice. These include the alteration of the immune landscape of humans by prior and current co-infections including the influence of immune responses to existing liver stage *Plasmodium* parasites [197]. However there has been some success in modelling co-infection scenarios in mice and measuring how immune responses to *Plasmodium* are influenced when co-infections are present (for examples see [11,198]). In addition, there are likely effects of the human biting rate (HBR) which would alter amounts of mosquito saliva exposure [199,200,201] and has been shown to influence *Plasmodium* infection in mice [202]. The entomological inoculation rate (EIR) may also differ and be associated with a varying number and/or multiplicity of *Plasmodium* infection in an individual [203]. Without use of mouse models of blood stage infection where each aspect can be dissected individually, it would be virtually impossible to determine the relative influence each of these environmental factors has on immune responses mounted to blood stage *Plasmodium* infection.

## 5. Mechanisms of Anti-Parasite Immunity: What Have We Learnt about Control of iRBCs from Mouse Models of *Plasmodium* Infection?

Successful control of intraerythrocytic *Plasmodium* parasites requires a robust cellular and humoral immune response that generates broadly-reactive antibodies. Rodent malaria models of *Plasmodium* erythrocytic infection have been instrumental in revealing some of the mechanisms governing the cellular immune responses to *Plasmodium* blood stage parasites, as well as spatial information related to immune responses generated in different organs where *Plasmodium* iRBCs sequester. It is challenging to obtain this level of information from human infections where the main available source for immune analysis is the peripheral blood. Here we discuss some of the main findings from use of rodent models of *Plasmodium* blood stage infection.

### 5.1. Invasion Blocking Is a Key Mechanism of Anti-Parasite Antibodies for the Control of iRBCs

The importance of humoral immunity in host defense against *Plasmodium* infection was first demonstrated in rhesus monkeys [204] and later in *P. falciparum*-infected children [205,206] when passive transfer of immune sera limited parasite growth and symptoms associated with the disease. These observations formed the basis of the hypothesis that there is a requirement for sustained antibody production in the control of *Plasmodium* blood stage of infection where the clinical manifestations of the disease occur.

The possible effector functions of these antibodies have been elucidated with careful in vitro culture studies. These range from recognition and uptake of iRBCs by phagocytes [207,208,209], blocking of parasite adhesion and invasion [210], to inhibition of parasite growth [211]. The targets of these antibodies are numerous and involve proteins expressed on the surface of merozoite required for RBC invasion such as merozoite surface protein 1 (MSP-1) or apical membrane antigen 1 (AMA) [212,213] as well as variant surface antigens such as *Plasmodium falciparum* erythrocyte membrane protein 1 (PfEMP-1) [214]. Positive correlations between the breadth, as well as magnitude, of the antibody response and successful control of iRBCs [212,213,215] provide further evidence of the importance of the humoral response in controlling *Plasmodium* iRBCs.

The relative contributions of these mechanisms to parasite control are hard to assign in humans; mouse models of infection have been instrumental in identifying the importance of invasion blocking as a key mechanism of iRBC control in vivo. Studies using FcγR−/− mice which are deficient in the FcγR used by phagocytes to detect IgG-opsonized iRBCs demonstrate that IgG-dependent phagocytosis is not a key mechanism of control of iRBCs, at least in the avirulent *P. yoelii* XNL model [216]. This conclusion is supported by a recent study whereby in vivo tracking of a single generation of labeled iRBCs of either *P. chabaudi* or *P. yoelii* adoptively transferred into mice demonstrated that parasite-specific IgG does not affect the rate at which iRBC are cleared, but rather it limits the progression of the iRBC to a new RBC by blocking invasion [210]. This is not a surprising finding when most IgG was reactive to merozoites found within schizonts, the terminal stage of iRBCs prior to release of merozoites that will infect new RBCs. The observation that infection of mice with *P. yoelii* XNL line becomes lethal in a B cell-deficient host [11,217] does not differentiate the role of IgG from other isotypes. There is a growing appreciation for the role that IgM plays in the control of iRBCs. IgM may mediate antibody dependent phagocytosis through the Fcμ receptor, although this receptor is expressed only on B cells in mice [218]. It is also possible that complement-mediated lysis of opsonized iRBCs could contribute to parasite control as shown in *P. falciparum* infections [219,220] although the effects of complement depletion has been shown to be minimal in the *P. chabaudi* AS mouse model [221].

It is important to note that antibodies do not appear to be an absolute requirement to control all species of *Plasmodium* infection in mice. Unlike in *P. yoelii* XNL infections, B cell-deficient mice infected with *P. chabaudi* are able to control acute infection via antibody-independent mechanisms [217,222]. Depletion of γδ T cells in B cell-deficient mice following *P. chabaudi* AS infection led to exacerbated parasitemia, indicating a more critical role for γδ T cells in cell-mediated immune response against *P. chabaudi* [223]. Thus, mouse models of *Plasmodium* infection indicate that some immune mechanisms of iRBC control may be differentially important for different species or clones of *Plasmodium*.

### 5.2. Plasmodium Blood Stage Infection Leads to the Development of Memory B Cells That Respond during Secondary Challenge Infection

Humoral immune responses against malaria develop slowly, inefficiently and wane over time in the absence of reinfection [224,225,226]. Antibodies are derived from antibody-secreting cells (ASCs) that are comprised of plasmablasts and plasma cells. These cell types are generated from a specialized compartment called the germinal center (GC) in secondary lymphoid tissue [227]. ASCs can also be found out-with the B cell follicles of the spleen. Mouse models of infection have been instrumental in demonstrating that this early response may have some protective qualities [228]. Memory B cells (MBCs) and long-lived plasma cells (LLPC) offering protection against re-challenge infections are also thought to develop in the GC after infection but some evidence suggests that these are heterogeneous populations and some sub-populations of MBCs may form prior to GC formation [229,230,231]. Given that secondary lymphoid tissue is not readily accessible for study in *Plasmodium*-infected humans, mouse *Plasmodium* infections have allowed dissection of cellular responses in humoral immunity to malaria. The investigation of B cell responses to blood stage *Plasmodium* has typically utilized both *P. chabaudi* AS and *P. yoelii* XNL. Despite differences in the importance of antibodies for iRBC control between these species, the cellular mechanisms underlying B cell responses to *Plasmodium* blood stage parasites appear similar regardless of which species was used to initiate infection [232,233,234].

The cellular basis underpinning the lack of efficacious long-lived humoral responses to *Plasmodium* in those living in endemic areas is still incompletely understood. Indeed, it has been shown that a large proportion of memory B cells in *P. falciparum* infection are IgM+ with IgG+ memory B cells developing with age [235]. IgM+ memory B cells in malaria harbor somatically hypermutated B cell receptors [97] suggestive of affinity maturation and can develop into plasma cells that secrete IgM neutralizing antibodies that have high invasion-blocking capability against *P. falciparum* in vitro [236], suggesting that they may play a key role in controlling iRBCs.

Following infection with *P. chabaudi*, *Plasmodium*-reactive memory B cells and plasma cells can be detected over eight months post-primary infection [97,99]. Upon secondary infection with homologous parasites, a more rapid production of IgG isotypes can be observed [99] indicating recall responses are active and functional. B cell tetramers that bind to MSP-1 have been used to show both class-switched IgG+ as well as somatically hypermutated IgM+ memory B cells participate in recall responses [97]. The utility of IgM+ memory B cells in the memory response to malaria has been demonstrated in the *P. chabaudi* mouse model of malaria where IgM+ B cells were the dominant MBCs expanding on challenge infections leading to the early protection against re-infections [97]. Work in the *P. yoelii* XNL [111] and *P. berghei* ANKA [237] models have taken these results further demonstrating participation of IgM+ memory B cells in splenic secondary germinal centers upon challenge [111] with upregulation of the necessary CD80 and CD73 co-stimulatory molecules resulting in differentiation into antibody secreting cells and the secretion of iRBC-reactive antibodies. Thus, mouse models of malaria have demonstrated that IgM+ memory B cells are a critical player in the secondary responses to malaria.

One of the key mechanisms underpinning an impaired memory B cell response may be related to apoptosis induced by blood stage infections. MSP-1 vaccinated BALB/c mice infected with the lethal *P. yoelii* YM strain led to ablation of memory B cells and LLPCs, including those that developed prior to vaccination with MSP-1 or unrelated antigens [238]. These data suggest that, although memory B cells and LLPCs can develop to blood stage infection, more lethal *Plasmodium* infections may have a deleterious effect on these cell subsets via induction of apoptosis, albeit by an unknown mechanism.

### 5.3. Development of Functional Anti-Plasmodium Blood Stage GC Responses

While there is evidence of the formation of GCs in individuals with malaria, there are some indications that GC reactions might not be optimal during human *Plasmodium* infection [239]. Mouse models have been instrumental in demonstrating that fully functional GCs can develop in a primary blood stage *Plasmodium* infection leading to protective B cell responses. In GCs, follicular helper T (Tfh) cells interact with B cells and help push differentiation of B cells into plasma cells (short-lived and long-lived) and memory B cells [227]. The *P. yoelii* mouse model of infection has been used to show that B cells are the primary cell type expanding Tfh cells [240]. Upon expansion, IL-21, one of the major Tfh cell-derived cytokines, has been shown to be important in the development of robust and durable class-switched B cell responses following blood stage infection with *P. chabaudi* AS and *P. yoelii* XNL [232]. Disruption of IL-21-derived signals on B cells led to a diminished level of *Plasmodium*-specific antibodies and resulted in increased parasitemia which was correlated with a deficiency in the development of plasma cells and memory B cells [232]. Furthermore, Tfh-deficient CD4^Cre^xBcl6^fl^ animals or SAP−/− deficient animals were unable to clear chronic infection with *P. chabaudi* AS [95] demonstrating that, although the establishment of chronic infection appears to be antibody-independent, antibodies are critical for control of chronic infection.

During *Plasmodium* infection GCs form in the context of innate-derived inflammatory responses as well as during responses to existing *Plasmodium* infections, particularly in higher transmission areas where simultaneous multiclonal infection is common [12,13]. Mouse models of *Plasmodium* infection have demonstrated that B cell priming of Tfh cells in the spleen after blood stage *Plasmodium* infection is dampened by type 1 interferon via downregulation of T-cell expressed Inducible CO-Stimulator (ICOS) and interruption of ICOS-ICOSR signaling between GC Tfh cells and GC B cells, respectively [234]. The interaction between ICOS-ICOSR is critical for Tfh cell development against blood stage *Plasmodium* infection in mice [241,242]. Furthermore, upregulation of the inhibitor molecules Programmed Death-Ligand 1 (PD-L1) on antigen-presenting cells and Lymphocyte Activation Gene-3 (LAG-3) [243] on T cells negatively regulate the development and function of Tfh cells. IL-6 also plays a role in Tfh cell differentiation in blood stage *Plasmodium* infection, albeit IL-6R signaling appears to be more important for plasma cell development [233]. Thus, Tfh cell development and the ensuing GC reactions are highly dynamic processes controlled by positive and negative molecular regulators. These can be soluble mediators such as cytokines, but also cellular mediators such as Cytotoxic Lymphocyte Associated Antigen-4 (CTLA4)-expressing T follicular regulatory cells [244] that downregulate B cell responsiveness or reactions outside of the follicle such as the rapid of expansion of extrafollicular plasmablasts that deplete the nutrients required for cells participation in germinal center reactions with the follicles [110].

A key feature of the immune response in *Plasmodium*-infected individuals is the induction of a strong production of pro-inflammatory cytokines, with IFN-γ a defining cytokine. *P. falciparum*-induced IFN-γ in human infection has been shown to drive the expansion of T-box Expressed in T cells (T-bet)+ CD21-CD27- atypical B cells [245] which upregulate inhibitory receptors. In some [245], but not all [246], studies atypical B cells appear to have reduced functionality with respect to antibody production. Atypical B cells develop in both the *P. chabaudi* AS and the *P. yoelii* XNL models of *Plasmodium* infections [132,146] making these mouse models a crucial tool in dissecting these contrasting observations in human studies. The expansion of atypical B cells in acute febrile *P. falciparum* infection are transcriptionally distinct from activated B cells or classical memory B cells and able to interact with Tfh cells to differentiate into antibody secreting cells [246]. Mouse models of non-lethal *Plasmodium* infection have been used to define the function of atypical B cells in vivo, particularly with respect to their contribution to the memory B cell compartment that remains upon resolution of acute *Plasmodium* infection. Using a transgenic mouse that harbored antigen-specific B cells with BCRs that react to MSP-1 atypical B cells were considered to be short-lived disappearing upon resolution of infection [132]. However using Fc Receptor-Like 5 (FCRL5) as a marker of long-lived memory B cells [132] it has also been shown in a secondary challenge infection in the *P. chabaudi* model that FCRL5-expressing memory B cells have a robust recall response and that some of these cells adopt an atypical phenotype in a T-bet-independent fashion [146].

Although there is evidence that IFN-γ both supports [112,145] or impairs [247,248,249] GC B cell responses in mouse models of *Plasmodium* infection, the effects are likely contextual. T-bet intrinsic expression in B cells, induced by signaling from the IFN-γ receptor is needed for IgG2c isotype class switching during *Plasmodium* blood stage infection and also enhances affinity maturation [145]. This IFN-γ likely comes from IL-21/IFN-γ expressing Tfh (Tfh1) cells [98]. Although first described in the periphery of *P. falciparum*-infected individuals [239], rodent models of malaria have been instrumental in demonstrating the lineage and function of Tfh1 cells [98] with interferon-mediated signaling via Interferon Regulatory Factor 3 (IRF3) supporting a developmental shift away from Tfh cells to Th1 cells [250]. More recent studies have comprehensively dissected the intracellular signals governing plasticity of the Tfh/Th1 cell phenotype in CD4+T cells responding to blood stage *Plasmodium* infection [251]. Accordingly, molecules that down-regulate T-bet-mediated IFN-γ secretion in B cells, such as IL-10 [252], promote humoral responses to blood stage *Plasmodium* infection [247]. T cell-derived IL-10 can also act directly on B cells early on in infection, influencing B cell survival, their interactions with Tfh and ultimately the formation of germinal centers [253].

There is still much to be learned regarding the factors that regulate the development of B cell responses to blood stage *Plasmodium*. The discovery of a novel population of NK1.1 T cells supporting antibody production from short-lived extrafollicular plasma blasts [254] demonstrates the complexity in the development and control of humoral responses to blood stage *Plasmodium* infections. The main rodent models utilized in investigating humoral responses to malaria involve the species *P. chabaudi* and *P. yoelii* due to their non-lethal phenotype in many backgrounds of mice, including C57BL/6. However, modelling the role of antibodies in severe malaria has been accomplished using *P. berghei* ANKA infections normally employed for immunopathogenesis studies. One study observed that the pro-inflammatory mediators that enhance the onset of pathology associated with severe malaria also affect the development of efficacious humoral immune responses through inhibition of Tfh cell differentiation and consequently compromised GC reactions [248]. With the development of B cell tetramers [97] and BCR-transgenic mice [132] to identify malaria-reactive B cells, mouse models of malaria will be needed for moving forward the analysis and definition of factors that influence a robust and efficacious humoral response with the precision required to identify more relevant metrics to signify efficacious candidate vaccines against malaria that elicit a long-lived protective response.

### 5.4. The Importance of Innate Immune Cells in Control of iRBCs

Antibody-mediated control of parasites via blocking of invasion is not the only immune mechanism of iRBC control. Ample data have been gathered on human *Plasmodium* infections clearly demonstrate functional activity of innate cells against iRBCs. The contribution of innate immune responses to *P. falciparum* in the Fulani tribe in sub-Saharan Africa has been attributed to their greater resistance to infection compared with more susceptible sympatric ethnic groups [255] and innate responses to *P. falciparum* in CHMI studies have been associated with subsequent control of both iRBCs and clinical symptoms [256]. In *P. falciparum* blood stage infection, innate cells of the myeloid lineage [105,257,258], neutrophils [259,260], natural killer (NK) [28,261,262,263,264] and γδ-T cells [265] have all been shown to neutralize iRBCs. Correlations of innate cell function with parasitemia or clinical symptoms have suggested the importance of these cells in control of iRBCs. However, mouse models have played a key role in deciphering how innate cells modulate adaptive responses and exert protection against the blood stages of *Plasmodium* in the context of the global response.

Studies of isolated antigen-presenting cells from human peripheral blood mononuclear cells (PBMCs) and cell lines derived from human myeloid lineages were initially used in combination with cultured *Plasmodium*-iRBCs from *P. falciparum* lines to investigate how iRBCs are recognized by the immune system. The biological significance of initial studies demonstrating a role for Pattern Recognition Receptors (PRRs) such as Toll-Like Receptors (TLRs) in the recognition of iRBCs, merozoites and products of schizogony such as GlycosylPhosphatidylInositol (GPI) [266] has only been possible using in vivo mouse models of malaria. There are several PRRs that recognize *Plasmodium*-iRBCs and these have been reviewed by Gowda and Wu [267]. Here we will focus on the contribution of mouse models to understanding the significance of GPI-TLR2 recognition and DNA/hemozoin-TLR9 recognition in the immunology of blood stage infections.

The inflammatory nature of GPI was shown using purified from *P. falciparum* but it was the injection of this molecule into mice that demonstrated that GPI-induce inflammatory responses may influence malarial symptoms [268]. Complementary studies blocking responses to GPI in mice immunized with the glycan portion of this molecule were protected from *P. berghei* ANKA-induced experimental cerebral malaria (ECM) [269]. In vitro macrophage cultures were used to demonstrate a receptor-mediated mechanism for the effects of *P. falciparum*-derived GPI [270] and to identify TLR2 in a heterodimer with TLR1 as the principal PRR in GPI recognition [271]. However it was infection of TLR2−/− mice with the non-lethal *P. chabaudi* AS line [272] or the non-lethal *P. yoelii* XNL line [273] that indicated there was very little impact on control of circulating iRBCs and the pathogenesis of infection. This is in contrast to a stark pathological role in experimental cerebral malaria (ECM) where, in agreement with antibody-mediated GPI blocking, TLR2−/− mice were protected from death from neurological symptoms during *P. berghei* ANKA infection [274]. GPI-mediated TLR2 signaling may be more pathogenic in this system given the effects of GPI signaling on endothelial cells [266], and the pathogenic role this may play in areas of iRBC sequestration and accumulation such as the brain in CM/ECM.

Similarly, mouse models have been instrumental in determining the role of innate sensing of parasite DNA and hemozoin, an insoluble crystalline by-product of hemoglobin digestion [275], on the immunology and pathogenesis of acute *Plasmodium* blood stage infections. It was initially demonstrated in 2004 that *P. falciparum* schizont extract contained a TLR9 ligand [276], classically thought to be unmethylated CpG motifs in pathogen DNA [277]. However, it has subsequently been shown that *Plasmodium*-derived DNA complexed with protein [278] or hemozoin [279] is able to signal through TLR9. The role of hemozoin in *Plasmodium* infection is complex: the *P. chabaudi* model suggests detrimental effects on splenic DCs that have internalized hemozoin crystals with respect to limited ability to activate T cell effector function in the spleen [280], possibly due to the induction of anti-inflammatory cytokines as shown in *P. yoelii* XNL model [273]. The consequences of this can be seen in *P. yoelii* XNL infection of TLR9−/− mice which have higher parasitemia [273], although control of *P. chabaudi* AS appears to be less affected [272]. This could be explained by the effects of hemozoin and TLR9 on B cells and antibody production. With respect to parasite control *P. yoelii* XNL is more dependent on antibody-mediated control of iRBCs than *P. chabaudi* AS [11]. More recent work in the *P. yoelii* model suggests that DNA sensing through TLR9 promotes the development of autoreactive Tbet+ B cells that may produce antibodies that recognize components of uninfected RBCs contributing to malarial anemia [281]. As for lethal infection and similar to TLR2, TLR9 sensing is a critical pathogenic factor in the development of ECM in the *P. berghei* ANKA model [274].

In addition to identifying the significance of PRR recognition of different *Plasmodium*-derived molecules during *Plasmodium* infections, mouse models of malaria have been instrumental in dissecting the relative importance of different cells from the myeloid lineage in control of iRBCs. Circulating monocytes are able to phagocytose *P. falciparum* [207,282] and *P. vivax* [283] iRBCs in both an opsonic and non-opsonic [284] manner. The *P. chabaudi* AS model has been used to demonstrate a significant contribution of monocytes [85] compared to neutrophils [86,87] in control of iRBCs. In agreement with these data, neutrophils also do not seem to be a dominant cell type involved in the control of iRBCs in non-lethal *P. yoelii* XNL infections [106]. However, neutrophils have been shown to play an important role in the *P. berghei* ANKA model where iRBCs are less controlled when neutrophils cannot make Neutrophil Extracellular Traps (NETs) [285]. Thus, it seems that differences in the function of myeloid cells may exist amongst rodent parasite species that may be related to parasite life cycle preferences such as infection of reticulocytes (*P. yoelii* XNL) over normocytes (*P. chabaudi* AS) or differences in inflammatory potential from iRBCs of different species.

Along a similar line, mouse models of malaria have been instrumental in understanding the contributions of NK cells and γδ-T cells with regard to control of iRBCs and the pathogenesis of infection. Both NK cells and γδ-T cells have been shown to be able to target *Plasmodium*-iRBCs. NK cells are able to directly recognize iRBCs to produce IFN-γ [286,287] and have been shown to confer protection in *P. falciparum* infection via cytokine production and ADCC that subsequently inhibits *P. falciparum* growth in RBCs [261,263]. However, NK activation has been shown to depend on accessory cells of the myeloid lineage [288] as well as T cells [289]. Infection of humanized mice with *P. falciparum* confirmed in vitro observations of contact-dependent NK cells in elimination of iRBCs [28]. Furthermore, using the non-lethal *P. yoelii* XNL mouse model of *Plasmodium* infection it has been shown in an in vivo setting that iRBCs induce activation of NK cells via synergistic effects of IL-18 and IL-12 to induce the expression of CD25 and IFN-γ production [290].

In acute malaria infection, an increase in the γδ-T cell numbers correlate with protection from high parasitemia [89,265]. However repeated exposure to malaria has been shown to lead to a decrease in circulating γδ-T cell numbers, particularly the subset that expresses the Vδ2^+^ chain of the γδ TCR [265,291,292]. Although it is not possible to define the role of the Vδ2^+^γδ-T cell subset in mice due to the absence of the Vδ2 chain in mice, in general γδ-T cells produce IL-21 and IFN-γ that may enhance humoral immune response against blood stage infection [293]. γδ-T cells produce IFN-γ, granzymes and granulysin that collectively inhibit parasite growth in a contact-dependent manner [88]. γδ-T cells displaying CD16^+^ Vδ2^+^ TCRs are able to respond to opsonized *P. falciparum* iRBCs through engagement of CD16 receptors [294] facilitating Vδ2^+^ T cell effector function with respect to ADCC cytotoxicity and in removal of iRBCs by phagocytosis [294,295]. CD16^+^ Vδ2^+^ T cells are shown to exhibit some of the features of NK cells and data also suggests they are more cytolytic than their CD16^−^ Vδ2^+^ T cell counterparts [294]. In addition, Vδ2^+^γδ-T cells appear to induce contact-dependent lysis of iRBCs via granulysin [295].

Mouse models of malaria have been used to assess the relative contributions of NK cells and γδ-T cells with respect to the control of iRBCs. Despite an evident early expansion of NK cells in the spleen upon infection with *P. chabaudi* [140] and the contribution activated NK cells play in ramping up the inflammatory response during *P. chabaudi* infection [296], NK cells only have a small impact on control of parasitemia during primary infection [223,297,298]. On the other hand, γδ T cells have been shown to exert significant control of iRBCs during acute infection with *P. chabaudi* AS, exceeding that mediated by antibodies [223]. γδ-T cells can also express M-CSF that protect against recurrence of *P. chabaudi* parasitemia in mice at the later stage of the infection [89]. In line with these findings, depletion studies in *P. chabaudi adami* 556KA-infected mice found a more prominent role for γδ T cells compared to NK cells in controlling iRBCs [223], a finding supported by a second study in *P. yoelii*-infected mice which found no significant role for NK cells in parasite control [105].

These data illustrate the power of undertaking in vivo experiments in uniform settings such as in laboratory mice where the contribution of different immune cells can be parsed out in a way that is not easy to undertake in culture studies or human infection studies, an exception being controlled human malaria infections [299,300]. As heterogenous innate cell populations become better defined with advances in technologies such as single-cell sequencing, mouse models of malaria will be instrumental in defining how cell sub-populations of innate cells enhance iRBC lysis and phagocytosis in a temporal setting and in relation to unfolding and established adaptive responses.

## 6. Immunopathogenesis of Malaria and Clinical Immunity

While sterile immunity preventing *Plasmodium* infection does not commonly occur, people living in malaria endemic regions ultimately develop clinical immunity that protects against symptoms associated with *Plasmodium* blood-stage infection. Clinical immunity to malaria is characterized by reduced parasitemia and attenuated inflammatory responses [301,302]. As such, people who develop clinical immunity to malaria often carry *Plasmodium* iRBCs asymptomatically with a low-grade pro-inflammatory immune response that limits blood stage parasite replication.

Based on human studies, clinical immunity has long been thought to center on the acquisition of strong immunomodulatory mechanisms that fine tune the inflammatory response necessary for control of the parasite burden while controlling the inflammation-induced pathology. Clinical symptoms of malaria are driven by Th1 inflammation characterized by IFN-γ, a cytokine known to be important in the development of immune effector mechanisms including high affinity class-switched anti-parasite antibody [245,303] and activation of phagocytes [304]. The main, but not only, sources of IFN-γ found in *P. falciparum* infection include Th1 cells, cytolytic CD8 T cells, NK cells and γδ T cells [304], in particular Vδ2^+^ γδ T cells [265], as high production of pro-inflammatory cytokines by Vδ2^+^ γδ T cells has been shown to protect from infection by *P. falciparum* in children living in a high transmission setting. Analysis of T cell responses after controlled human malaria infection (CHMI) with *P. falciparum* demonstrated that higher blood stage parasitemia was associated with an expansion of T regulatory (T reg) cells that express CD25 and FoxP3 after schizogony from the liver [300], suggesting downregulation of the inflammatory response supports parasite replication in the blood stage.

At the same time, systemic inflammation appears to correlate with the pathogenesis of malaria. Higher IFN-γ production from γδ T cells diminishes clinical immunity in response to subsequent infections with *P. falciparum* [265], presumably due to inflammation-induced pathogenesis. As such, decreased Vδ2^+^ T cell numbers, and an upregulation of immunoregulatory markers such as Tim-3 and CD57 on γδ T cells, is associated with clinical immunity to malaria [265,291]. Along the same lines, the identification of CD4 T cells producing both IFN-γ and IL-10, termed Tr1 (Foxp3^-ve^ regulatory) cells, have been identified in *P. falciparum*-infected individuals [305] and associated with uncomplicated disease in children. Indeed, a longitudinal analysis of children from an endemic region of Mali indicates that a recent exposure to *Plasmodium* changes the cytokine profile of a subsequent *Plasmodium*-infection, specifically upregulation of IL-10 in children with persistent asymptomatic infection [301]. These data suggest that IL-10 offers protection from clinical symptoms of malaria. Furthermore, there are multiple correlative studies suggesting a protective role of transforming growth factor- β (TGF-β) against clinical symptoms of malaria [306,307,308]. Collectively, these data support a role for inflammation-suppressing cytokines in protecting against the pathogenesis of malaria.

Mouse models of non-lethal malaria have been used to confirm the importance of inflammatory responses in contributing to the control of iRBCs, including the contribution of CD8 T cells which can recognize *P. yoelii XNL*-infected erythroblasts that express peptide-loaded MHC-I on their surface [309] and kill them via Fas-FasL dependent cytotoxicity [310] and ganulysin-mediated mechanisms of cytotoxicity [48]. A transgenic mouse with Pb-I CD8+ T cells reactive to the 60s ribosomal protein L6 (RPL6) has provided evidence that CD8 T cells can be primed in the liver stage and boosted when there is a shared antigen with blood stage malaria [127,128]. The ability of CD8 T cells to contribute to parasite control has been replicated in the *P. chabaudi* AS model [311,312,313] but ultimately these data suggest that this contribution is not essential for the control of parasitemia in several mouse models of malaria blood stage infection [314] and CD8 T cells in cannot confer protection without the help of CD4 T cells [315].

Given the well-defined pathologies that can be measured in the mouse models of malaria there have been several studies confirming the importance of immunomodulatory cytokines such as TGF-β [93] and IL-10 [94,109] against malaria pathogenesis. Nonetheless, IL-10 and TGF-β are both pleiotropic cytokines with several possible sources. Thus, the main contribution of studies in mouse models of *Plasmodium* infection has been the ability to dissect the roles of these pleiotropic cytokines throughout the course of the infection, as well as identify the most potent sources of these cytokines mediating clinical immunity to the blood stages of *Plasmodium* infection. For example, comparison of the lethal (*P. yoelii* XL) and non-lethal (*P. yoelii* XNL) strains of *P. yoelii* revealed that early production of TGF-β (within 24 h) in lethal infection is associated with delayed IFN-γ and TNF-α production, leading to uncontrolled parasite growth and 100% fatality [113]. This was in contrast with a later (day 5 post-infection) production of TGF-β in non-lethal infection which was associated with reduced parasitemia and resolution of the infection [113]. In a similar vein, the timing of IL-10 during the progression of malarial disease seems to be crucial for control of severe immunopathology [316].

With regards to IL-10, the absence of which turns a non-lethal *P. chabaudi* AS infection into a lethal one [94], mouse models have been used to determine that this cytokine is essential in the control of pathogenic TNF-α production [94,109]. Mouse models have challenged the notion that these immunoregulatory cytokines were produced by classical CD4+ CD25+ FoxP3+ T reg cells. Early studies comparing lethal *P. yoelii* XL and nonlethal *P. yoelii* XNL infections demonstrated a similar expansion and activation of Treg cells following infection with these two strains, indicating that the early activation of Treg cells does not contribute to the virulence [109]. Indeed, studies of TGF-β induction by *P. yoelii* indicated that the main producers of TGF-β were in fact CD8^+^ CD25^+^ T reg cells.

On the other hand, the main source of IL-10 has been found in both the *P. chabaudi* [317] and *P. yoelii* [109] models to come not from classical T reg cells, but rather from FoxP3-ve T cells that have been shown to simultaneously-produce IFN-γ [317]. The presence of IL-10/IFN-γ Tr1 cells has been shown in human infection [318] but it is in mouse models that the production of IL-10 and IFN-γ in Tr1 cells has been shown to be dependent on IL-27 signaling [317,319]. IL-10 production by Tr1 cells (CD4^+^ CD25^−^ Foxp3^−^ CD127^−^) was able to down-regulate iRBC-induced pro-inflammatory responses [109] resulting in the increased growth of parasitemia in non-lethal *P. yoelii* XNL infection. It has been subsequently shown that the immune regulatory role of IL-10-producing Tr1 may differ between primary and secondary infection in blood stage *Plasmodium* infection: IL-10 may exert a more suppressive effect on the innate immune system, specifically MHC-II expression on APCs, during primary infection while it suppresses the adaptive system, specifically expansion of antigen-experienced memory CD4 T cells, in secondary infections [320]. The use of double IFN-γ-YFP^+^ and IL-10-GFP^+^ reporter mice have indicated that following resolution of primary infection, the stability and potential of CD4^+^ IFNγ^+^ IL-10^+^ T cells to become memory is limited [320], in part because they exhibit an exhaustion phenotype and are generally unresponsive at the early stage of secondary infection.

### 6.1. Organ-Specific Pathologies

Severe cases of malaria are associated with organ dysfunction which can be caused by sequestration of iRBCs via vascular adhesion [321] and trapping of iRBCs in the capillaries due to reduced deformability [322,323,324]. With the advent of luciferase expressing constructs, the rodent *Plasmodium* parasites have been shown to sequester in different organs in mice [56,118,325,326]. Given the relative inaccessibility of human organs from patients experiencing severe malaria syndromes, the rodent models of *Plasmodium* infections have been instrumental in dissecting immunopathological mechanisms associated with localized inflammation from sequestered and accumulated iRBCs [45,56,119]. Models of particular malaria-associated syndromes can be achieved using different combinations of rodent parasite species and mouse backgrounds (Table 2). Here, we focus on how mouse models of blood stage malaria have contributed to our understanding of the immunological underpinnings of three of the most well-studied sequelae of malaria: Severe Malarial Anemia (SMA), Cerebral Malaria (CM) and Acute Respiratory Distress Syndrome (ARDS).

### 6.2. Mechanisms of Inflammation-Induced SMA

SMA in children is defined as a hemoglobin value < 5 g/dL and detectable parasitemia in the blood stream [327]. Although *Plasmodium* replication in RBCs results in physical destruction of the RBC, SMA is more likely caused by mechanisms that result in hemolysis [328] and clearance of both uninfected and iRBCs via erythrophagocytosis [329] in combination with disrupted erythropoiesis in the bone marrow [330]. The relative contributions and mechanisms underlying these different contributors to a reduction in circulating RBCs are difficult to assess in humans without splenic or bone marrow biopsies. Sequestration/accumulation of iRBCs in the inflamed bone marrow has been shown [331]. However, mouse models of SMA, principally the non-lethal *P. chabaudi* model, have been instrumental in demonstrating the underlying mechanisms of anemia and in dissecting the relative contributions of each.

Early work in using *P. chabaudi* as a model for SMA established that dyserythropoiesis in malaria may result from stalling of late erythroid progenitor cells [332] and be related to bone marrow inflammation, in particular production of the pro-inflammatory cytokines IL-12 [333] and macrophage migration inhibitory factor (MIF) [334]. A role for type 2 cytokines, specifically IL-4, has also been shown to suppress late erythroid progenitor cells [335]. Inflammation is likely derived from iRBCs that accumulate in the bone marrow, but early studies suggested that “malaria toxins”, free GPI anchors that are released during iRBC schizogeny [336], can directly lead to dyserythropoiesis [337,338]. Hemozoin has also been shown to induce anemia [339], demonstrating a contribution from parasite products in the suppression of erythrocyte production. Nonetheless, the density of circulating iRBCs is not necessarily related to level of anemia in the *P. chabaudi* model [58] suggesting that direct parasite destruction of RBCs during replication and release of inflammatory products during schizogony plays a more minor role in the severity of malarial anemia. Given the insoluble and persistent nature of hemozoin, the contribution of hemozoin may be cumulative over time during chronic infection.

Whilst existing data using rodent models of *Plasmodium* infection point to a direct suppression in the development of late erythroid progenitor cells via inflammatory cytokine induction, there may also be an indirect effect via cytokine modulation of erythropoietin produced by the kidney [340]. Other studies have investigated whether a defect in iron handling also contributes to suppression of erythropoiesis [341] and how this may be reversed [342]. Other than production of new RBCs during the process of erythropoiesis, removal of both infected and uninfected circulating RBCs has been shown to occur in the liver via erythrophagocytosis in rodent infections [343]. The removal of uninfected RBCs via autoimmune antibody-dependent mechanisms has been suggested in human malaria [344], and these include antibodies that recognize band 3, an anion transporter that mediates RBC flexibility which is the most abundant erythrocyte surface receptor [345], phosphatidyl serine (PS) that becomes exposed on the surface RBCs [346] and spectrin, a cytoskeletal molecule important for maintaining the stability and structure of the cell membrane [347]. Autoantibodies to band-3 can occur naturally but appear to be elevated in *Plasmodium*-infected individuals [348,349].

The contribution of anti-band-3 antibodies to malarial anemia is uncertain and may depend on the infecting species of *Plasmodium*. However, anti-PS antibodies have been demonstrated to contribute to RBC removal in the *P. yoelii* XNL model [350] providing a mouse model in which to test the significance of this mechanism with respect to malarial anemia. Plasma cells producing anti-PS antibodies in the *P. yoelii* model have been identified as CD11c+ Tbet+ atypical B cells that are induced in response to DNA sensed by TLR9 and IFN-γ [281]. Work in the *P. chabaudi* model has been used to show that malaria appears to prime B cell clones that already exist, producing autoantibodies with a strong IgG response to band-3 and spectrin [351]. Thus, in addition to defining relative contributions of autoantibodies in the severity of malarial anemia relative to other mechanisms, mouse models of malarial anemia allow interrogation of the cellular mechanisms underlying how autoantibodies arise.

Moving forward, mouse models of blood stage *Plasmodium* infection will be instrumental in determining how iRBCs interact in the bone marrow niche [352] and the mechanisms by which extramedullary erythropoiesis are established in an attempt to remedy diminished circulating RBCs, particularly in the red pulp of the spleen [353,354]. It will also be pertinent to determine the degree to which removal of uninfected RBCs by autoantibodies impacts on the levels of anemia.

### 6.3. T Cell-Mediated Breakdown of the Blood-Brain Barrier in Cerebral Malaria

Pediatric cerebral malaria (CM) is almost always fatal when not treated with antimalarials, and still has mortality rates between 15% and 20% with treatment [355]. The initiation of CM is thought to occur as a result of sequestration and adherence of iRBCs to the brain vasculature leading to disruption of the blood-brain barrier (BBB), a complex of cells and extracellular structures that regulates the exchange of molecules between the blood and the central nervous system. BBB disruption occurs upon activation of brain microvascular endothelial cells. Although markers of vascular activation can be measured in the bloodstream of individuals with CM [356], the mechanism by which the BBB breaks down is poorly understood, in part due to the paucity of brain tissue availability from victims of CM and other control groups for comparison. As such, rodent models of CM are essential to enable cellular mechanisms of BBB breakdown in CM to be elucidated and rationally targeted therapeutically.

Infection of C57Bl/6 mice with *P. berghei* ANKA recapitulates many of the features that characterize human CM (Table 2) and is a commonly used model described as ECM [357]. Compared to some of the other ECM models, *P. berghei* ANKA infection of C57BL/6 mice does not rely on extremely high parasitemia to cause disease. Infected mice usually die between 6 and 10 days after infection [358] with accumulation of iRBCs to the brain microvasculature [359] and the activation of brain endothelial cells [360,361]. Human studies suggest that both host and parasite factors mediate the development of CM in *P. falciparum*-infected children. As such there is some debate on the utility of the rodent ECM model, particularly since there are some differences in the expression of parasite adhesion molecules such as CD36 on human brain microvascular endothelial cells [362,363] compared with mouse [364]. Furthermore *P. berghei* ANKA lacks PfEMP-1, a ligand of both Intercellular Adhesion Molecule-1 (ICAM-1) and Endothelial Protein C Receptor (EPCR) on brain endothelial cells, and this interaction is thought to facilitate sequestration [365]. *P. berghei* ANKA iRBC accumulation in organs relies on the expression of the Schizont Membrane-Associated Cytoadherence protein (SMAC) on the surface of *P. berghei* ANKA iRBCs [364]. Nonetheless, there is ample microscopic evidence, particularly using 2-photon techniques, of iRBC accumulation in brain microvessels with some sequestration of iRBCs on the endothelial lining [115,366,367]. Experiments with luciferase-expressing *P. berghei* ANKA strain clearly show focused accumulation in the brain, particularly in the brain stem and olfactory bulb [40,368,369,370,371,372].

Inflammation related to sequestered iRBCs is thought to be a central facet of the pathogenesis of CM, and is necessary for the pathogenesis of ECM. Neuroinflammation often involves the production of TNF-α but ablation of TNF-α using Etanercept did not reduce the mortality rate of pediatric CM [373]. Data using the ECM model of CM concurs with this finding whereby infection of TNF-α deficient mice still die from BBB breakdown in the same time frame as intact animals [374]. Indeed, ECM has been critical in demonstrating the importance of lymphotoxin-α (LT-α), rather than TNF-α, in mediating breakdown of the BBB [374,375].

In addition to TNF-α and LT-α, interferons are a key facet of the neuroinflammatory response to iRBCs. *Plasmodium* parasites are known to induce type 1 IFNs (IFN-I) which, depending on context, have the capacity to both suppress and activate innate and adaptive immune cells, promote pro-inflammatory cytokine production and enhance parasite clearance. Both IFN-I subtypes a signal through the heterodimeric IFNAR functioning in both an autocrine and paracrine manner. The binding of IFN-I to IFNAR induces a signal cascade that initiates the transcription of interferon stimulated genes (ISGs). Host genetic variation can lead to differences in gene regulatory regions of the IFNAR1 subunit of IFNAR. The development of cerebral malaria in children has previously been associated with a variant of IFNAR1 associated with a higher expression of IFNAR1 [376,377,378,379]. This suggests that Type 1 interferon signaling is a pathogenic event [376] and is a finding supported by studies in ECM [141,380].

Similarly, there have been associations with polymorphisms in the IFN-γ receptor [381] and lower levels of plasma IFN-γ [382] with development of CM suggesting that IFN-γ is protective. This is in agreement with polymorphisms in the IFN-γ gene promoter which are associated with increased transcription of IFN-γ and protection from CM [382]. The ECM model relays a different story: IFN-γ has been shown to be necessary for death to occur [358,383]. IFN-γ derived from CD4 T cells [384] leads to trafficking of pathogenic CD8 T cells to the brain [358,384] and cross-presentation of merozoite-derived epitopes on major histocompatibility complex (MHC)-I [360] for recognition by infiltrating parasite-reactive CD8 T cells and BBB disruption. In ECM, IFN-γ leads to upregulation of adhesion molecules on brain microvascular endothelial cells enhancing the adhesive properties of *P. berghei* ANKA iRBCs [385]. The reason for the apparent difference in the role of IFN-γ in BBB disruption is unknown, but may be related to the differences exerted on parasite control mechanisms initiated by IFN-γ in the context of a more chronic setting than that studied in the ECM model.

One of the significant breakthroughs in our understanding of the immunological underpinning of CM from the ECM model was the demonstration that CXCR3 [386] and CCR5 [387]-dependent CD8 T cell infiltration into the brain is necessary for disruption of the BBB [114]. In mice IFN-γ, including that secreted by NK cells [386], induces production of CXCR3- and CCR5-responsive chemokines in the neurovascular unit thus facilitating recruitment of pathogenic CD8 T cells and other immune cells to the Central Nervous System (CNS) [388]. Whilst initial studies on human autopsy samples indicated a cellular infiltrate that was largely devoid of CD8 T cells [389,390], suggesting a potential fundamental difference in the etiology of BBB breakdown between human CM and mouse ECM, more in depth studies from pediatric CM victims in Malawi have provided evidence that CD8 T cells do infiltrate the brain [390,391], and this increase in CD8 T cells is correlated to density of iRBC sequestration [391]. As such, CD8 T cell involvement in human CM can no longer be ruled out.

The mechanisms by which CD8 T cells mediate breakdown of the BBB via effects in the endothelium are still poorly understood but the ECM model has been critical in elucidating some of the parameters by which this occurs. It has been shown in ECM that lytic molecules perforin and granzyme B [392,393] are essential components in this process. Evidence of apoptosis in brain endothelial cells can be observed in autopsy samples of pediatric CM cases [388,394] as may be expected via the lytic action of incoming primed CD8 T cell recognition of cross-presenting brain endothelial cells. Although apoptosis can also be seen in brain sections of *P. berghei* ANKA in addition to 2-photon microscopy [367], it is minimal. Furthermore, infected mice do not have a significantly increased cleaved caspase compared with naïve mice [393]. Whilst other mechanisms of brain microvascular endothelial cells such as necrosis, ferroptosis and pyroptosis have not been extensively investigated, these data do suggest that perforin and granzyme are acting through non-cell death-inducing pathway that disrupts the BBB. In this regard findings in the ECM model are similar to Theiler’s murine encephalomyelitis in mice, another model of CD8 T cell dependent disruption of the BBB, where perforin but not FasL is required to mediate vascular leakage and death [395].

The findings that CD8-derived lytic enzymes might not act via induction of apoptosis leads to the idea that they may act to disrupt the BBB via another mechanism. They may have a role in downregulating tight-junction and adherens-junction proteins which normally enable endothelial cells to dynamically control the passage of solutes and other molecules across the BBB [396]. Disassembly and downregulation of junction proteins on brain microvascular endothelial cells has been observed in both pediatric CM autopsy samples [397] and in ECM [40,366]. In ECM CD8 T cell-degranulation may induce downregulation of junction proteins via release of perforin [393,398] which could augment expression of vascular activation-induced molecules such as the tyrosine kinase receptor EphA2 which has been shown to mediate the loss of tight junctions on both human and mouse brain microvascular endothelial cells [40]. In the Theiler’s murine encephalomyelitis model, leakage and downregulation of tight junction proteins occurs before an increase in apoptosis markers [398]. Thus, the timing of BBB breakdown in ECM and CM relative to initial *Plasmodium* infection may be important in the interpretation of ECM studies as applied to CM.

Given that BBB disfunction is a feature of both CM in humans and ECM in mice, the rodent model of ECM is a crucial tool in unravelling the most important mechanisms that lead to fatal pathogenesis. Endothelial cells are only one player in the neurovascular unit that also includes mural cells (pericytes), astrocytes and microglia [399]. It is hard to discount the potential role of these accessory cells in disassembly of inter-endothelial junction proteins in CM given that astrocytes and microglia are both activated in ECM [400,401,402] and the known role they play in regulation of BBB integrity. Indeed, molecules secreted from these cells upon activation can be measured in the cerebral spinal fluid of children with CM [403] and pediatric autopsy samples demonstrate activation of microglia and astrocytes in fatal CM [404]. The mechanisms by which these accessory cells become activated, and the mechanisms which control endothelial cell junction protein modulation in CM, remain to be discovered. Given the difficulty in studying these cells, it is likely that the ECM model will be instrumental in disentangling the cellular and molecular basis of endothelial cell junction disassembly.

Identification of immunodominant epitopes from *P. berghei* ANKA allows for more in-depth studies on the characteristics of the CD8 T cells that are pathogenic in ECM. In addition to the glideosome associated protein 50 (GAP50)_40–48_ epitope in the context of H2-D^b^ [360], two further immunodominant epitopes in the *P. berghei* ANKA model have been identified: bergheilysin protein (Pb2)_592–599_ and replication protein A1 (F4_199–206_) [405] both in the context of MHC Class I H2-K^b^. This work has allowed the development of tetramers to track *Plasmodium*-reactive CD8 T cells in vivo and opens up the possibility of developing TCR transgenic animals, such as the PbI transgenic mice that recognize parasite-derived 60S Ribosomal protein L6 (RPL6)[127] in several *Plasmodium* species, including *P. falciparum*, that can be used to investigate the mechanisms by which CD8 T cells might mediate BBB breakdown. In addition, the ECM model is likely to be important in the identification of possible avenues for therapeutic targeting, such as possible IL-33 administration to induce anti-inflammatory cytokine expression and the expansion of anti-inflammatory macrophage and regulatory T cell populations [406] or IL-15 complex treatment to protect BBB leak by expanding a population of IL-10 producing NK cells [407].

### 6.4. Mechanisms of Malaria-Associated Acute Lung Injury (MA-ALI) in Malaria-Associated Acute Respiratory Distress Syndrome (MA-ARDS)

Pulmonary complications arising from *Plasmodium* infection can occur with all species but in particular upon infection with *falciparum*, *vivax* and *knowlsei* species. This is a syndrome of severe malaria resulting in up to 40% mortality even with treatment [20]. Though more common in adults infected with *vivax* malaria, in children, MA-ARDS can often present along with cerebral complications [22]. MA-ARDS is characterized by increased permeability of pulmonary capillary endothelial cells and alveolar epithelial cells, with Pulmonary Edema (PE), hypoxia [20] and in some cases fibrosis [408,409,410]. Most data related to the pathogenesis of MA-ARDS and MA-ALI comes from post-mortem studies of lung tissue from adult fatalities of *Plasmodium* infection showing apoptosis of alveolar cells [21]. However, the immunological mechanisms underlying MA-ARDS and MA-ALI in *Plasmodium* patients are relatively understudied and poorly understood.

Pulmonary vascular activation is thought to arise in response to the sequestration of iRBCs resulting in inflammation in the lung microvasculature [17,21] characterized by expression of TNF-α [22], Von Willebrand Factor (VWF) and ANGiopoietin-2 (ANG2) [411]. However, sequestration of iRBCs is likely to occur via a different suite of adhesion molecules upregulated on the pulmonary vasculature compared with the BBB. For example, EPCR expression which is a key molecule mediating adhesion of iRBCs on brain microvascular endothelial cells [412] has been found to be significantly downregulated on pulmonary vasculature endothelial cells in those who have succumbed to MA-ARDS [22] compared with those dying of other malaria-related syndromes.

Some studies employ the *P. berghei* ANKA strain used to study ECM by virtue of the fact this strain sequesters in the lung [413,414] and the ultrastructure of the infected lung looks similar to postmortem samples from victims of MA-ARDS [116]. However, MA-ARDS and MA-ALI are more commonly studied using infection with the NK65 strains of *P. berghei* [45]. The advantages of the *P. berghei* NK65 models are that they do not appear to result in neurological manifestations of infection and have higher ARDS clinical scores, than *P. berghei* ANKA infection [45]. *P. berghei* NK65 iRBCs accumulate in the lung vasculature, with an increase in VWF expression [415] as also found in human *Plasmodium* infections. There are two primary strains of *P. berghei* NK65 used for studies in the pathogenesis of MA-ARDS and MA-ALI: the Edinburgh strain (*P. berghei* NK65E) and the New York strain (*P. berghei* NK65NY). Possibly due to slower growth of iRBCs due to the predilection of *P. berghei* NK65NY to infect reticulocytes, the *P. berghei* NK65NY does not recapitulate MA-ARDS despite sequestering in the lung tissue. However, the Edinburgh strain results in rapid death of C57BL/6 mice from days 6–10 post-infection and recapitulates features of MA-ARDS seen in humans such as extensive neutrophil infiltration, an increase in pulmonary VWF expression [415] and an increase in protein concentration in lungs [45]. *P. berghei*-NK65E has been used to demonstrate the critical role of VWF in alveolar leakage [415].

In mice, MA-ARDS and MA-ALI appear to have similarities regarding the underlying pathogenesis of ECM. Studies on MA-ARDS/ALI using *P. berghei* ANKA infection have demonstrated that IFN-γ, upregulation of chemokines [416] and functioning CD8 T cells are all necessary for lung sequestration of iRBCs and pulmonary edema [417]. In addition, pulmonary vascular leak and BBB breakdown are dependent on the presence of platelets [41]. Unlike the BBB where molecules such as ICAM-1 and EPCR have been shown to play a key role, sequestration in the lung appears to be more dependent on the scavenger receptor CD36 [364]. There also appears to be a difference in the importance of myeloid cells with marked infiltration of neutrophils [418] and monocytes [419] to the lung which, at least for monocytes, appear to play a key role in controlling iRBC numbers.

The suite of *P. berghei* strains available to study this syndrome of malaria will be of some help in the interpretation of pulmonary autopsy samples from patients who have died of MA-ARDS and MA-ALI, a necessary endeavor given the lack of other tractable options to study this in *Plasmodium*-infected humans.

## 7. Conclusions

In summary this review has highlighted the utility of the rodent models of *Plasmodium* infection with regards to understanding the immunology of blood stage malaria. Several models exist although none completely recapitulate all aspects of malaria. However, this reflects the heterogeneity of this disease. Choosing the correct model to investigate specific aspects of this disease is essential in order to be able to extrapolate to human *Plasmodium* infections. There is still a plethora of key outstanding questions that remain in the field of blood stage immunology of malaria (see Outstanding Questions box). With the advent of genetically-modified rodent *Plasmodium* strains and an ever-increasing catalog of genetically-modified and transgenic mice in addition to SPF genetically diverse mice available through the CC and DO mouse resources, these questions can only be answered with the employment of mouse models of blood stage malaria. Analysis of spatial aspects of anti-malarial immune responses can only be studied in the context of infection rather than employing in vitro studies. The mechanisms at play in immunologically-driven organ-specific pathogenesis of malaria can only be holistically studied using a whole-organism approach and in organs that are not readily accessible in humans. For all of these reasons, the tractable rodent models of malaria described here will be a critical tool with respect to answering these outstanding questions in the field of blood stage malaria. In turn, the information gained will be instrumental in the rational design of novel immunologically-based therapeutic strategies that are badly needed in the fight against this disease.
